# Identifying Clinical and Genetic Characteristics of Spinal Muscular Atrophy Patients and Families in Saudi Arabia

**DOI:** 10.7759/cureus.46452

**Published:** 2023-10-04

**Authors:** Alaa Alghamdi, Shaikhah AlDossary, Wala Abdulaziz Alabdulqader, Fawzia Amer, Mona Ali, Momen Almomen, Fouad Alghamdi

**Affiliations:** 1 Primary Care and Population Health, King Fahad University Hospital, Dammam, SAU; 2 Primary Care and Population Health, University College London, London, GBR; 3 Physical Therapy, King Fahad Specialist Hospital, Dammam, SAU; 4 Pediatric Neurology, King Fahad Specialist Hospital, Dammam, SAU; 5 Pediatric Neurology and Metabolic, Cairo University Children Hospital, Cairo, EGY; 6 Neurology, Neuroscience Center, King Fahad Specialist Hospital, Dammam, SAU; 7 Pediatric Neurology, Neuroscience Center, King Fahad Specialist Hospital, Dammam, SAU

**Keywords:** kingdom of saudi arabia (ksa), case series, carriers, mutation, sma

## Abstract

Introduction: Spinal muscular atrophy (SMA) is an inherited, neuromuscular disease characterized by the deterioration of spinal motor neurons, causing progressive muscular atrophy and weakening. It is an autosomal recessive disease with the mutation of the survival motor neuron 1 (SMN1) gene as a hallmark. Evidence suggests that the SMN2 gene modulates the severity of the disease. SMA is classified based on the maximum motor function achieved. This study aims to describe the genetic makeup and characteristics of an SMA cohort in the Kingdom of Saudi Arabia (KSA).

Methods: Data from families presenting with SMA children was collected between January 2018 and December 2020. Blood samples were collected from patients and family members. Genetic testing for SMA and mutations was performed at a European central lab.

Results and discussion: Seventeen families were enrolled in the study, including 52 children. Among 34 parents, 28 were carriers with heterozygous deletion (82.3%), one (2.9%) had no deletion detected by multiplex ligation-dependent probe amplification (MLPA) but had point mutation by sequencing, one (2.9%) had homozygous deletion and was symptomatic, three (8.8%) had no deletion or point mutation and were presumed to have 2+0, and one (2.9%) was not tested.

Conclusion: This study provides insight into the carrier mutational analysis of families with SMA disease manifestations in KSA. Further studies are needed to understand the burden and impact of SMA among the Saudi population.

## Introduction

Spinal muscular atrophy (SMA) is an autosomal recessive, inherited neuromuscular disease. It is characterized by progressive loss of motor neurons within the spinal cord, leading to muscle weakness and atrophy. Disruption of motor neurons interferes with the signals between neurons and muscles, leading to progressive weakness and wasting [1]. SMA is the second most common inherited disorder, with a global incidence of about one in 6,000 live births. Its carrier frequency is 1:40 for type I and 1:80 for the juvenile form (type III) [2]. SMA incidence in the Middle East is 40 times higher than that in the Western world ranging from 10 to 193 per 100,000 births. The increased rate of consanguineous marriages in the Middle East might have contributed to the high survival motor neuron-1 (SMN1) carrier frequency estimated at 1:20, compared to the global rate reaching up to 1:100 [3].

SMA patients present in four forms depending on the motor acquisition and the age of presentation. Infantile-onset SMA type I, also known as the Werdnig-Hoffmann disease, is the most common type accounting for 60% of all cases, and symptoms start before six months of age. SMA type II, or the intermediate form, starts to manifest between six and 18 months of age. In SMA type III, affected children develop symptoms after 18 months of age and can walk independently [4]. In SMA type IV, individuals develop symptoms after 21 years of age, with mild proximal muscle weakness, which typically does not affect ambulation and independent living. In addition to the neuromuscular manifestations, recurrent respiratory infections, scoliosis, and joint contractures are common complications of SMA. A comprehensive, multidisciplinary approach to SMA management prolongs patients’ lifespans and improves outcomes [5].

Deletion of the SMN1 gene on the long arm of chromosome 5 is the most common form of SMA [6]. SMN1 gene produces a protein called SMN1, which maintains the integrity of motor neurons. The absence of this protein causes progressive degeneration of anterior horn cells, leading to progressive muscle atrophy and paralysis. Another homologous gene, SMN2, is located on chromosome 5. Through alternative mRNA splicing, SMN2 produces a small amount of full-length, functioning SMN1 protein. Therefore, the number of SMN2 copies modulates the severity of the disease, with a higher number predicting a milder prognosis [3,7].

Biallelic pathogenic variants in SMN1 cause the most common form of SMA. Homozygous deletions of exon 7 of the SMN1 gene account for almost all typical SMA molecular diagnosis [8]. Less than 5% of SMA patients have a compound heterozygous mutation of SMN1 with a deletion in one allele and a subtle variation in the other allele [9]. Most heterozygous SMA carriers have one SMN1 copy in one of the alleles being quantitatively diagnosed. Exceptionally, carriers may have two SMN1 genes in cis and are called (2+0) carriers. Being indistinguishable from non-carriers (1/1), the (2+0) genotype could be a complicating SMA carrier diagnosis.

To our knowledge, few studies describing SMA's prevalence and carrier status in the Gulf region are present. Therefore, understanding the disease inheritance and demographics in the Kingdom of Saudi Arabia (KSA) is essential. This study is an observational analysis of phenotypes and genotypes of an SMA cohort seen in a tertiary care hospital in KSA, highlighting the familial genetic inheritance of the SMU genes and the frequency of silent 2+0 carriers.

## Materials and methods

Methodology

Patient Selection and Blood Collection

In this study, SMA patients presenting to King Fahad Specialist Hospital, Dammam, Saudi Arabia, between January 2018 and December 2020 were enrolled. Pediatric neurologists evaluated all SMA cases in King Fahad Specialist Hospital to ensure patients met the diagnostic criteria established by the International SMA Consortium. Dry blood spots were collected from all patients and family members.

The Ethics Committee of King Fahad Specialist Hospital reviewed and approved the study. All enrolled subjects fulfilling the diagnostic criteria signed written informed consent forms prior to blood sample collection.

Genetic Analysis

All 87 blood samples were sent to a central laboratory (Centogene Lab) in Germany for processing. At the Centogene Lab, genomic DNA was extracted from blood spots, and a multiplex-ligation-dependent probe amplification (MLPA) assay was performed for all collected samples. The SMN1 and SMN2 were simultaneously amplified by polymerase chain reaction (PCR) using the same primers. With unrevealing results, a follow-up SMN1 gene sequence was ordered to confirm the diagnosis. Once a genetic diagnosis of the index patient was established, MLPA testing for the corresponding patient’s parents and siblings was performed to identify SMN1 carrier status and SMN2 copy number. Patients who failed to show a homozygous absence of SMN1 exon 7 by MLPA were submitted to the sequencing of SMN1/SMN2 genes by next-generation sequencing (NGS).

Statistical analysis

Descriptive statistics were performed to examine the genetic makeup of 17 families with 87 children affected by SMA. Categorical variables were presented by counts and percentages. The analysis highlighted the genetic makeup of all families. Data from all families who underwent genetic screening were included in the full analysis set, which is the primary analysis population.

## Results

Over the study period, 87 individuals from 17 families encompassing 34 parents and 52 children were enrolled and included in the final analysis.

Description of SMN1 patients and genetic makeup

Among the enrolled patients, 23 children had no SMN1 copies. Two patients harbored one copy of the SMN1 gene and one point mutation and identified c.599dupT and p.Met200llesf*56. The median age of disease onset was two years (0.42-7 years) with a slight male predominance (56%). Type 3 SMA was the most identified SMA type among our population (Table 1). Among the 23 patients (92%) harboring zero copies of SMN1 (homozygous deletion), one (4%) was pre-symptomatically detected, four (16%) had type 1, six (24%) had type 2, and 12 (48%) have type 3. Only two (8%) compound heterozygous patients had one point mutation in one remaining copy of SMN1 and phenotypically behaved as type 1 SMA.

**Table 1:** Characteristics of SMA patients (categorical variables) SMA: Spinal muscular atrophy; SMN: Survival motor neuron.

			SMA type			
		N(%)	Pre-symptomatic	Type 1	Type 2	Type 3
Gender	Male	14 (56.0%)	1 (4.0%)	4 (16.0%)	2 (8.0%)	7 (28.0%)
	Female	11 (44.0%)	0 (0.0%)	2 (8.0%)	4 (16.0%)	5 (20.0%)
SMN1 copies	0	23 (92.0%)	1 (4.0%)	4 (16.0%)	6 (24.0%)	12 (48.0%)
	1	2 (8.0%)	0 (0.0%)	2 (8.0%)	0 (0.0%)	0 (0.0%)
SMN2 copies	2	6 (24.0%)	0 (0.0%)	5 (20.0%)	1 (4.0%)	0 (0.0%)
	3	8 (32.0%)	1 (4.0%)	1 (4.0%)	5 (20.0%)	1 (4.0%)
	4	11 (44.0%)	0 (0.0%)	0 (0.0%)	0 (0.0%)	11 (44.0%)
Type of mutation	Deletion	23 (92.0%)	1 (4.0%)	4 (16.0%)	6 (24.0%)	12 (48.0%)
	Point mutation	2 (8.0%)	0 (0.0%)	2 (8.0%)	0 (0.0%)	0 (0.0%)

**Figure 1:**
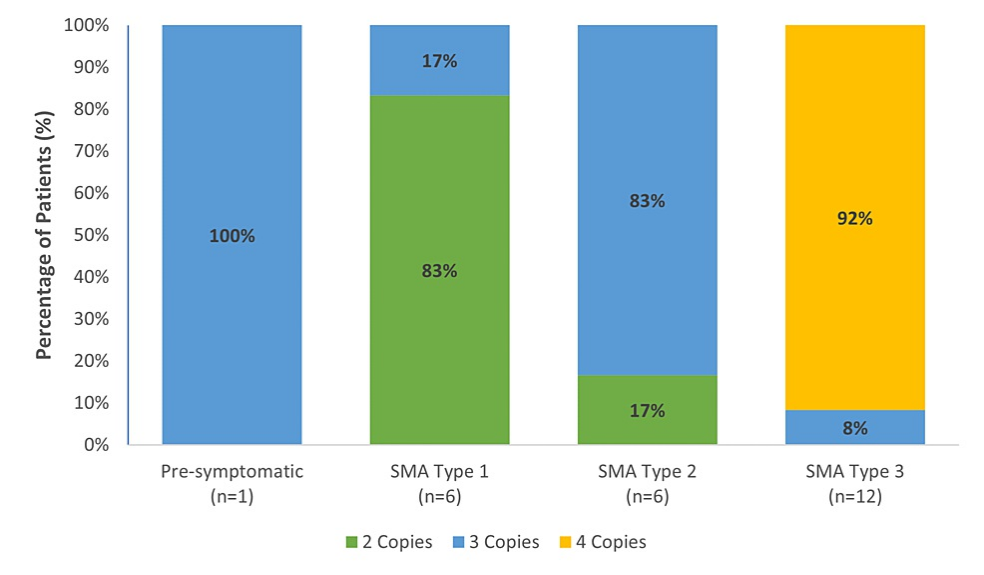
Percentage of spinal muscular atrophy disease type prognosis SMA: Spinal muscular atrophy.

Eleven patients (44%) harbored four copies of SMN2, all of whom had type 3 SMA. On the other hand, eight (32%) had three copies of SMN2 with one (4%) being pre-symptomatic, one (4%) had type 1, five (20%) had type 2, and one (4%) had type 3. Furthermore, only six patients (24%) had two copies of SMN2, five (20%) had type 1 SMA, and one (4%) had type 2 SMA. The number of detected copies of SMN2 varied among different SMA types. Type 1 SMA was the most common type to have two SMN2 copies. Most patients with type 2 had three SMN2 copies, while patients with type 3 SMA had the highest number of four SMN2 copies (Figure 1).

Description of SMN1 families and genetic makeup

Among the parents tested, 28 (82.3%) were carriers, one (2.9%) harbored a point mutation, one (2.9%) had zero copies of SMN1, and three (8.8%) were silent carriers, also known as (2+0) genetic makeup, indicating the presence of both SMN1 alleles on the same chromosome with its absence on the second pair. One parent did not undergo the screening. A total of 19 (36.5%) siblings were carriers with one copy of the SMN1 gene, nine (17.3%) were negative, seven had two SMN1 copies, one had four copies, and one had one copy.

Thirteen (76%) of all families had the typical genetic makeup for SMA disease, where each of the parents was a carrier with one SMN1 copy, and their offspring included either positive, carrier, or negative cases (Table 2). Eight families (2, 3, 7, 8, 10, 11, 12, and 16) showed 50% positive cases out of the total family children. In these families, six children were non-carriers, and four were carriers. All parents were carriers, two of whom (father in family 8 and mother of family 10) were found to be silent carriers. On the other hand, two families (9 and 17) showed 25% positive cases out of the total family children (Table 2).

**Table 2:** Examination of genetic makeup (SMN1) for each family SMN1: Survival motor neuron 1.

	SMN1 copies (test result)						
	Family member	Status	Number of SMN1 copies	Child number	Status	Number of SMN1 copies	% of +ve cases out of all children
Family 1	Mother	Carrier	1	Child 1	Positive	0	40%
				Child 2	Positive	0	
				Child 3	Carrier	1	
	Father	Carrier	1	Child 4	Carrier	1	
				Child 5	Carrier	1	
Family 2	Mother	Carrier	1	Child 1	Positive	0	50%
				Child 2	Positive	0	
	Father	Carrier	1	Child 3	Carrier	1	
				Child 4	Carrier	1	
Family 3	Mother	Carrier	1	Child 1	Positive	0	50%
	Father	Carrier	1	Child 2	Carrier	1	
Family 4	Mother	Carrier	1	Child 1	Positive	0	33.3%
				Child 2	Negative	2	
	Father	Carrier	1	Child 3	Carrier	1	
Family 5	Mother	Carrier	1	Child 1	Positive	0	33.3%
				Child 2	Negative	2	
	Father	Carrier	1	Child 3	Carrier	1	
Family 6	Mother	Positive	0	Child 1	Positive	0	60%
				Child 2	Positive	0	
				Child 3	Positive	0	
	Father	Carrier	1	Child 4	Carrier	1	
				Child 5	Carrier	1	
Family 7	Mother	Carrier	1	Child 1	Positive	0	50%
	Father	Carrier	1	Child 2	Negative	2	
Family 8	Mother	Carrier	1	Child 1	Positive	0	50%
	Father	Carrier (False negative)	2	Child 2	Negative	1	
Family 9	Mother	Carrier	1	Child 1	Positive	0	25%
				Child 2	Carrier	1	
	Father	Carrier	1	Child 3	Carrier	1	
				Child 4	Carrier	1	
Family 10	Mother	Carrier (False negative)	2	Child 1	Positive	0	50%
	Father	Carrier	1	Child 2	Carrier	1	
Family 11	Mother	Carrier	1	Child 1	Positive	0	50%
	Father	Carrier	1	Child 2	Carrier	1	
Family 12	Mother	Carrier	1	Child 1	Positive	0	50%
	Father	Carrier	1	Child 2	Carrier	1	
Family 13	Mother	Negative	3	Child 1	Positive	1	66.6%
				Child 2	Positive	1	
	Father	Carrier (False negative)	2	Child 3	Negative	4	
Family 14	Mother	Carrier	1	Child 1	Positive	0	100%
	Father	Carrier	1				
Family 15	Mother	Carrier	1	Child 1	Positive	0	40%
				Child 2	Positive	0	
	Father	Carrier	1	Child 3	Negative	2	
				Child 4	Negative	2	
				Child 5	Carrier	1	
Family 16	Mother	Carrier	1	Child 1	Positive	0	50%
				Child 2	Positive	0	
	Father	Carrier	1	Child 3	Carrier	1	
				Child 4	Carrier	1	
Family 17	Mother	Carrier	1	Child 1	Positive	0	25%
				Child 2	Negative	2	
	Father	Not done		Child 3	Carrier	1	
				Child 4	Negative	2	

Family 6 included an affected mother and a carrier father. This family had three out of five children (60%) affected with SMA, and the other two (40%) were carriers with one copy of SMN1.

Furthermore, family 8 consisted of a normal father (two SMN1 copies) and a carrier mother (one SMN1 copy) and had an offspring affected with SMA. This could be explained by the theory of (2+0) mutation. Although the father had two SMN1 copies, both copies could be present on only one allele.

Regarding family 13, the mother harbored three copies of SMN1 and tested normal. In addition, the father was a silent carrier with two SMN1 copies. Two out of the three children had point mutations. One of the two affected children tested positive with one copy of SMN1 and the other tested negative with four copies of SMN1. The unaffected sister had four SMN1 copies, which is an extremely rare condition. PCR and sequencing were conducted for the two affected children and identified the same point mutation in both children: c.599dupT and p.Met200llesf*56.

## Discussion

This study presents the first molecular and phenotypic analysis for Saudi patients with confirmed SMA and their families, using MLPA as a reliable method. The majority of SMA patients are linked to the 5q13 region; however, the complexity of this region results in various genetic rearrangements. Studies have shown that 95%-98% of SMA patients had homozygous deletion of both copies of the SMN1 gene. The remaining population is usually a result of compound heterozygosity with one intragenic mutation on one allele and SMN1 deletion on the other [10]. This was consistent in our study where 92% carried a biallelic deletion of the SMN1 gene, and 8% were compound heterozygous. Our cohort may have been liable to ascertainment bias as the severe form of the disease; type 1 is more fatal limiting this subtype access to our tertiary care clinic. This contrasts with the milder, non-life-threatening type 2 and type 3 [11].

In line with previous literature, our study showed that disease phenotype severity in SMA patients was inversely related to the number of SMN2 copies [5,12]. The majority of the enrolled SMA type 1 patients had two SMN2 copies, while the majority of SMA type 2 had three copies. Interestingly, our study showed that more than 90% of patients with SMA type 3 had four SMN2 copies. This is an unusually high percentage of patients; however, SMN2 copy numbers are not the only factor that influences the severity of SMA. Some SMA type 1 cases had three SMN2 copies and one asymptomatic patient had three copies. Mendonça et al. had similar observations, where most homozygous patients with SMA types 1 and 2 carried two copies (73%) and three copies (65%) of SMN2, respectively. However, most patients with type 3 SMA carried three or four copies of SMN2 [10]. On the other hand, this distribution was different in heterozygous patients. A similar correlation between SMN2 copies and SMA phenotype was also reported by Feldkötter et al. in 2003 and Calucho et al. in 2018 [12,5]. However, not all previous literature was consistent with the mentioned pattern [4]. Although the number of SMN2 copies is suggested to have a direct influence on the SMA phenotype and disease severity, other factors should be considered such as SMN1 mutations on both alleles, environmental factors, or other unknown factors. In addition, the impact of geographic distribution and racial differences should be considered and thoroughly studied. A previous study highlighted the inter-geographic variations in SMN2 copies among different populations. For instance, SMN2 copy number distribution differed between SMA patients from Mali, Nigeria, and Kenya. In addition, the percentage of African SMA patients with no SMN2 was significantly higher than European populations. This implies the importance of population-based national and regional SMA patient registries and studies to determine unknown factors influencing SMA patients.

**Figure 2:**
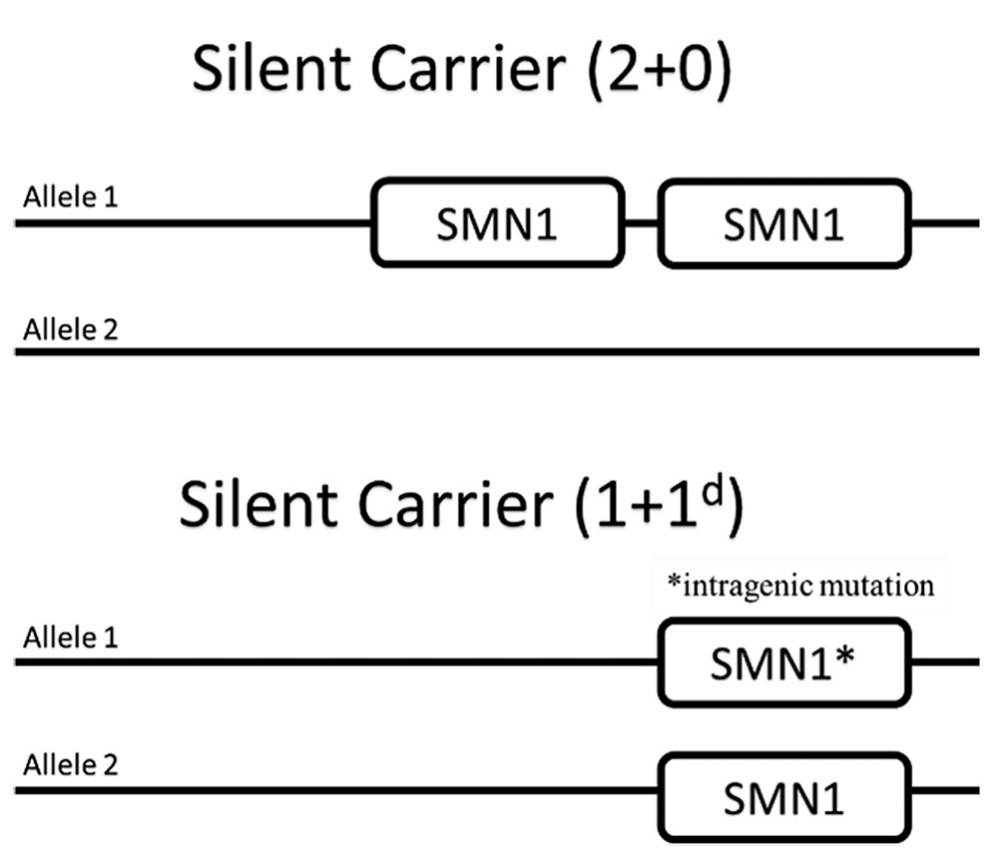
Silent carrier (2+0) of two copies of SMA1 on a single chromosome and silent carriers with one copy of SMA1 on both chromosomes with an intragenic mutation in one copy SMN1: Survival motor neuron 1; SMA: Spinal muscular atrophy.

In our study, 85% of the 34 parents were identified as carriers, having one SMN1 copy, when screening the families of genetically confirmed SMA patients. Applying deletion screening alone could be generally misleading in identifying SMA carriers. False-negative carrier status may result from either (i) identifying two SMN1 copies on one chromosome in carriers or (ii) missing intragenic SMN1 mutations in non-carriers. In addition to silent carriers, other possibilities can explain an unrevealing SMA parental carrier testing. First, carriers with (1 + 1d) genotype (where “d” designates intragenic mutation) have one healthy SMN1 copy on one chromosome and one intragenic mutant SMN1 on the other chromosome. Second, either parent might be a carrier of germ-line mosaicism, defined as a mutation exclusively found in the gonads but not in somatic cells. Third, parental somatic mosaicism may show a normal genotype ([1 + 1] genotype) in the somatic cells, which will prevent the detection of a genotype heterozygous for SMN1 deletion ([1 + 0] genotype) in the germ-line cells (Figure 2) [13-15].

Interestingly, three parents (9%) were identified to harbor two SMN1 copies in cis on one chromosome ([2+0] genotype). This frequency of (2+0) genetic makeup in parents of SMA patients was higher than that reported in some studies, which ranged between 4.3% and 4.6% of screened parents. The SMA parents in family 8 were both carriers; however, the father had two SMN1 copies, while the mother had one copy. We believe the SMA offspring in family 8 had zero SMN1 copies as a result of the father’s (2+0) genotype. The same situation existed in family 10; however, the mother was the SMA parent harboring two SMN1 copies and was believed to be the carrier parent with the (2+0) genotype. Family 13 had an unusual scenario, where the mother had three SMN1 copies and was tested as the only normal parent in our study. However, the father tested as a carrier while having two SMN1s. Even with the abundance of parental SMN1 copies, two offspring were identified as patients. The two offspring in this family were identified as type 1 SMA patients with an identified point mutation: c.599dupT and p.Met200llesf*56. Affected alleles with intragenic point mutations on the SMN1 are rare and are not detected by conventional analysis. To our knowledge, this point mutation is novel and has not been described before. Individuals with silent carrier (2+0) genomic status have a 50% chance to transmit their null allele to their offspring. Although challenging, premarital identification of such rare exceptions of SMA carriers is of utmost importance in Saudi society. Therefore, this descriptive study sheds light on the necessity of parental screening to identify silent carriers who present with duplicated SMN1 copies in a cis configuration.

This is the first study that reports the genetic inheritance of SMA in KSA. Further countrywide, multicenter studies are required to bridge the knowledge gap and raise awareness about SMA among local communities. The findings of this study provide healthcare workers and policymakers with preliminary data to understand the disease status in the region. However, one limitation of this study is that it was a single-center study limited by a small sample size. Furthermore, this study has an inherent bias as it was conducted in a highly consanguineous population, and its results may not represent the global SMA population.

## Conclusions

An essential finding in our study is the importance of including SMA carrier testing in the premarital testing program in the kingdom to identify silent carriers with (2+0) genotype, which has a relatively higher incidence in this population. The initiation of a newborn screening program might also solve the delay in diagnosing SMA and, therefore, decrease the severity and the burden of the disease. Additionally, screening might aid in the understanding of the clinical and molecular characteristics of SMA patients.
